# Supplementary material for: Molecular Mechanisms Underlying TNFα-Induced Mitochondrial Biogenesis in Human Airway Smooth Muscle
Source: Int J Mol Sci. 2023 Mar 17;24(6):5788. doi: 10.3390/ijms24065788 (PMC10055892; doi:10.3390/ijms24065788)
Supplement: Supplementary file 1 [file ijms-24-05788-s001.zip › ijms-2267624-supplementary.pptx]

## Slide 1
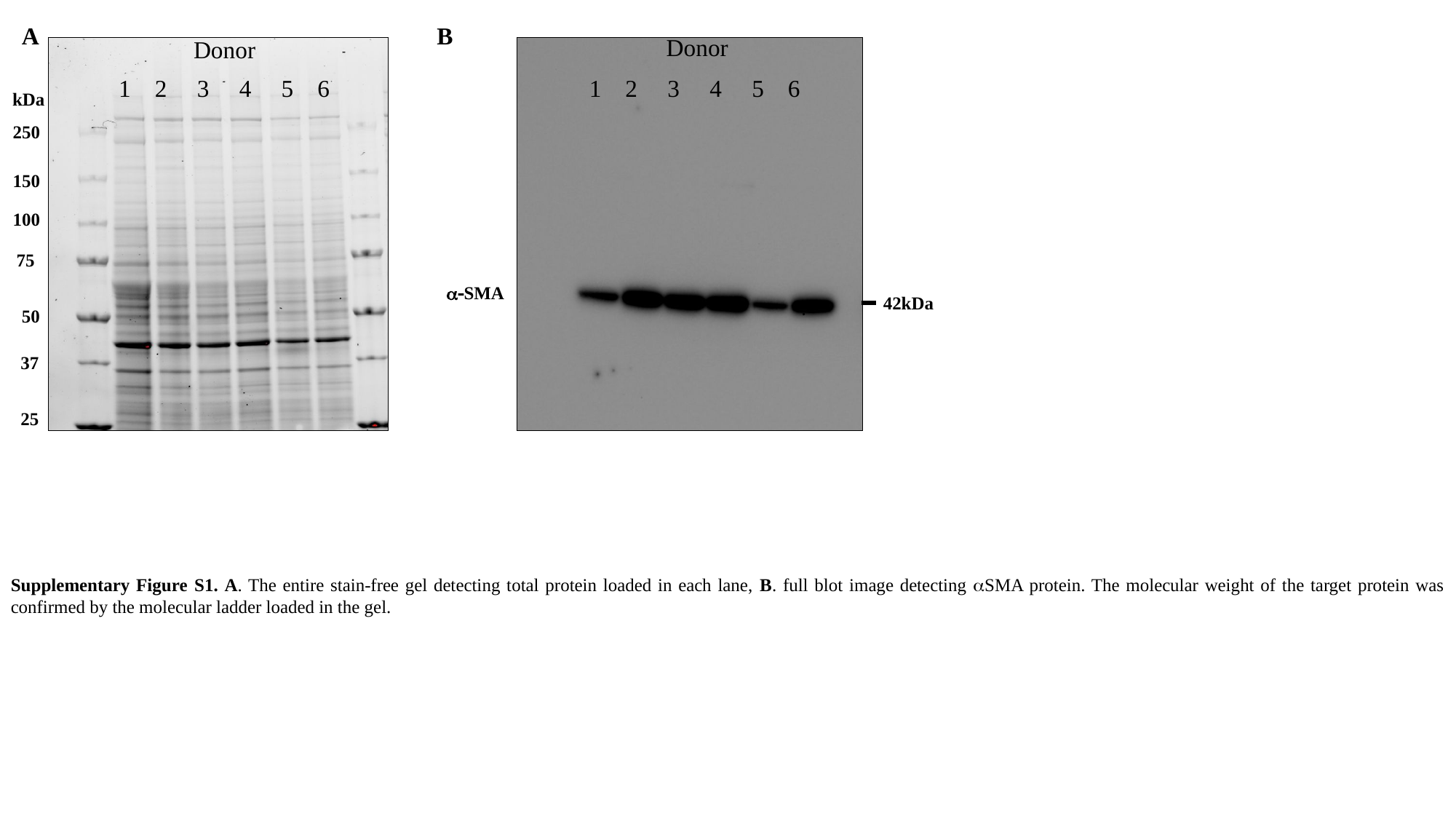

A
B
Donor
Donor
1 2 3 4 5 6
1 2 3 4 5 6
kDa
250
150
100
75
50
37
25
a-SMA
42kDa
Supplementary Figure S1. A. The entire stain-free gel detecting total protein loaded in each lane, B. full blot image detecting aSMA protein. The molecular weight of the target protein was confirmed by the molecular ladder loaded in the gel.

## Slide 2
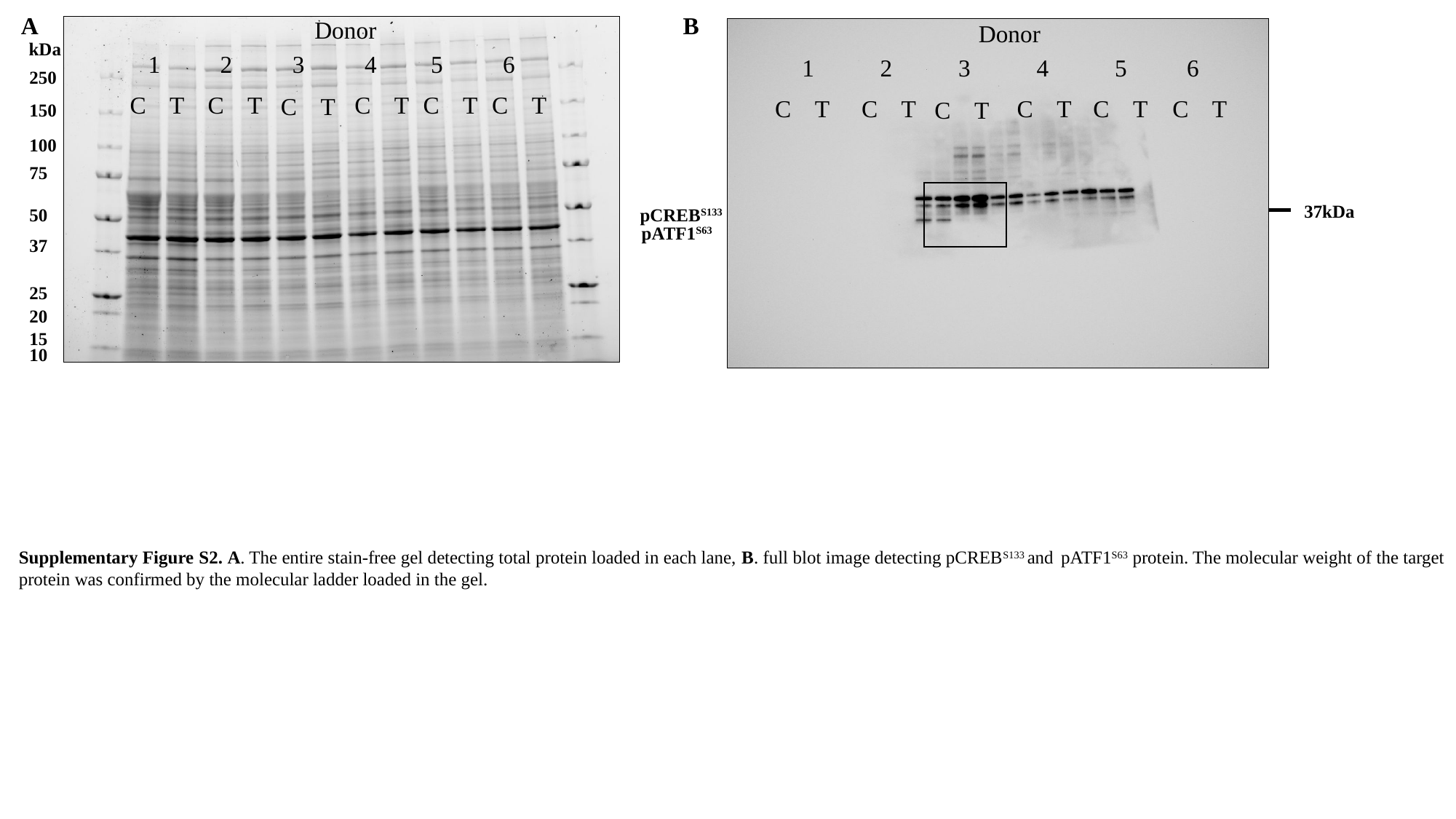

B
A
Donor
1 2 3 4 5 6
C T
C T
C T
C T
C T
C T
Donor
 1 2 3 4 5 6
C T
C T
C T
C T
C T
C T
kDa
250
150
100
75
50
37
25
20
15
10
37kDa
pCREBS133
pATF1S63
Supplementary Figure S2. A. The entire stain-free gel detecting total protein loaded in each lane, B. full blot image detecting pCREBS133 and pATF1S63 protein. The molecular weight of the target protein was confirmed by the molecular ladder loaded in the gel.

## Slide 3
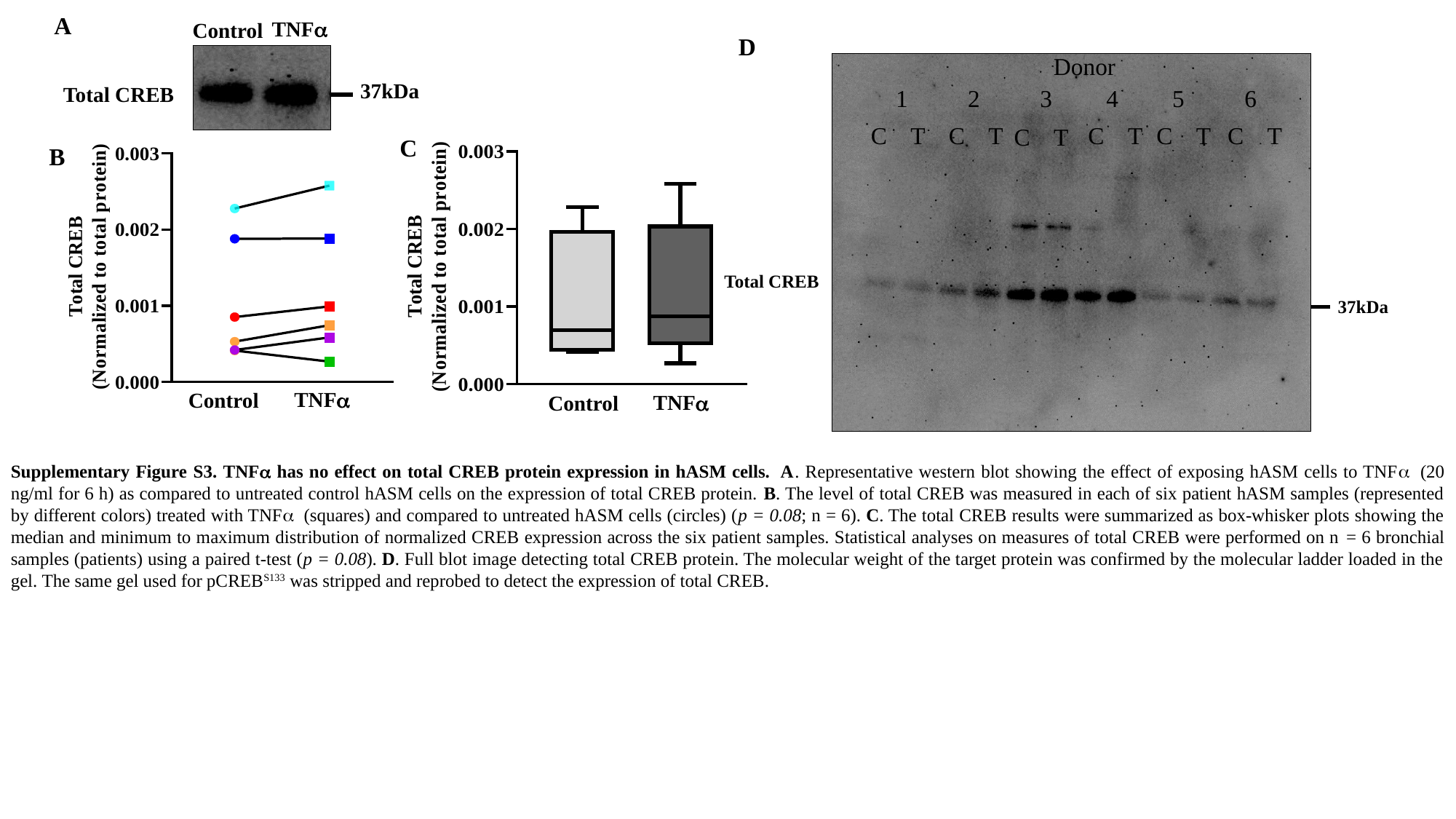

A
TNFa
Control
37kDa
Total CREB
D
Donor
 1 2 3 4 5 6
C T
C T
C T
C T
C T
C T
Total CREB
37kDa
TNFa
Control
TNFa
Control
C
B
Supplementary Figure S3. TNFa has no effect on total CREB protein expression in hASM cells. A. Representative western blot showing the effect of exposing hASM cells to TNFa (20 ng/ml for 6 h) as compared to untreated control hASM cells on the expression of total CREB protein. B. The level of total CREB was measured in each of six patient hASM samples (represented by different colors) treated with TNFa (squares) and compared to untreated hASM cells (circles) (p = 0.08; n = 6). C. The total CREB results were summarized as box-whisker plots showing the median and minimum to maximum distribution of normalized CREB expression across the six patient samples. Statistical analyses on measures of total CREB were performed on n = 6 bronchial samples (patients) using a paired t-test (p = 0.08). D. Full blot image detecting total CREB protein. The molecular weight of the target protein was confirmed by the molecular ladder loaded in the gel. The same gel used for pCREBS133 was stripped and reprobed to detect the expression of total CREB.

## Slide 4
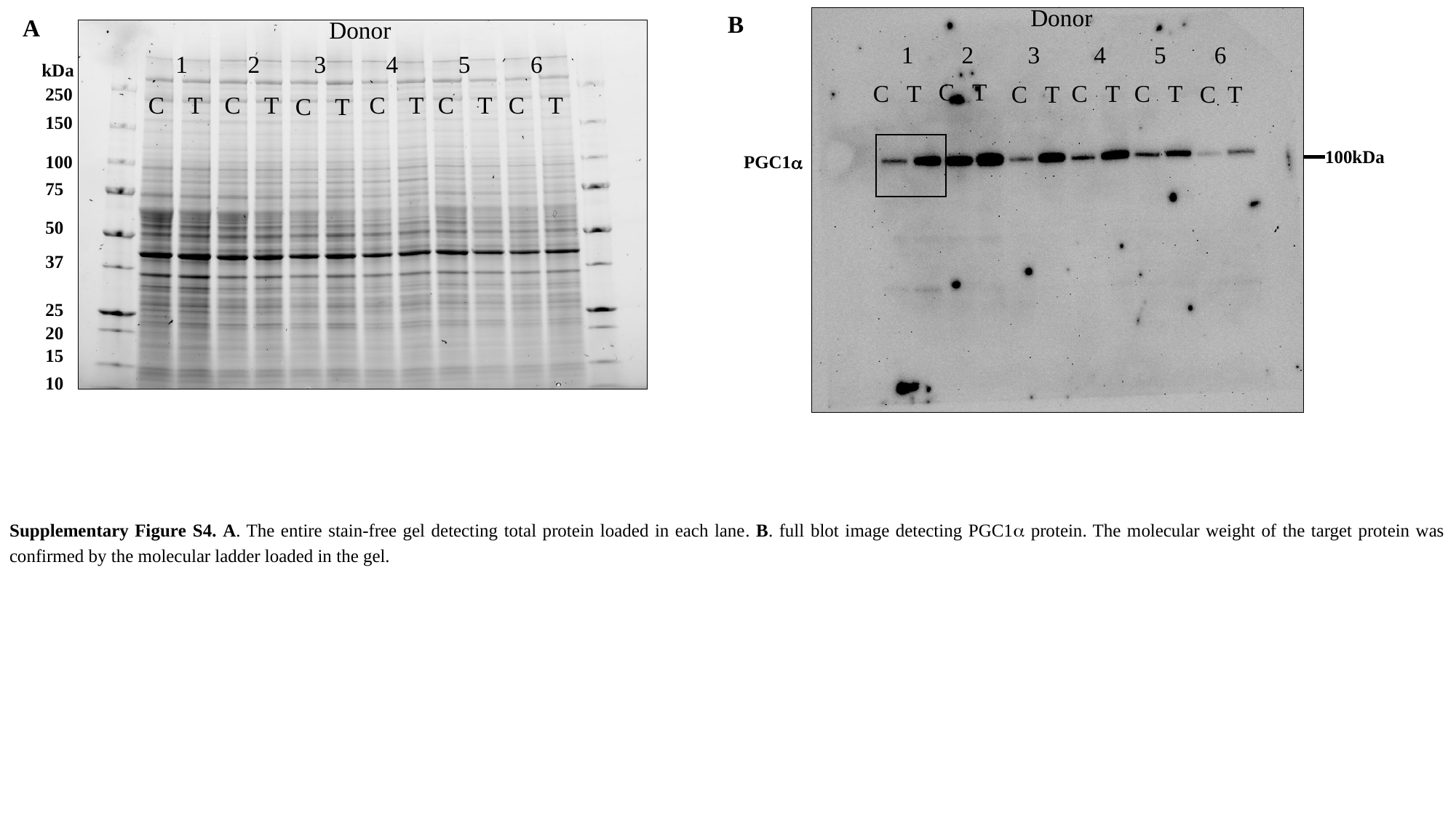

Patient
1 2 3 4 5 6
C T
C T
C T
C T
C T
C T
PGC1a
Donor
1 2 3 4 5 6
C T
C T
C T
C T
C T
C T
B
A
Donor
1 2 3 4 5 6
C T
C T
C T
C T
C T
C T
kDa
250
150
100
75
50
37
25
20
15
10
100kDa
Supplementary Figure S4. A. The entire stain-free gel detecting total protein loaded in each lane. B. full blot image detecting PGC1a protein. The molecular weight of the target protein was confirmed by the molecular ladder loaded in the gel.

## Slide 5
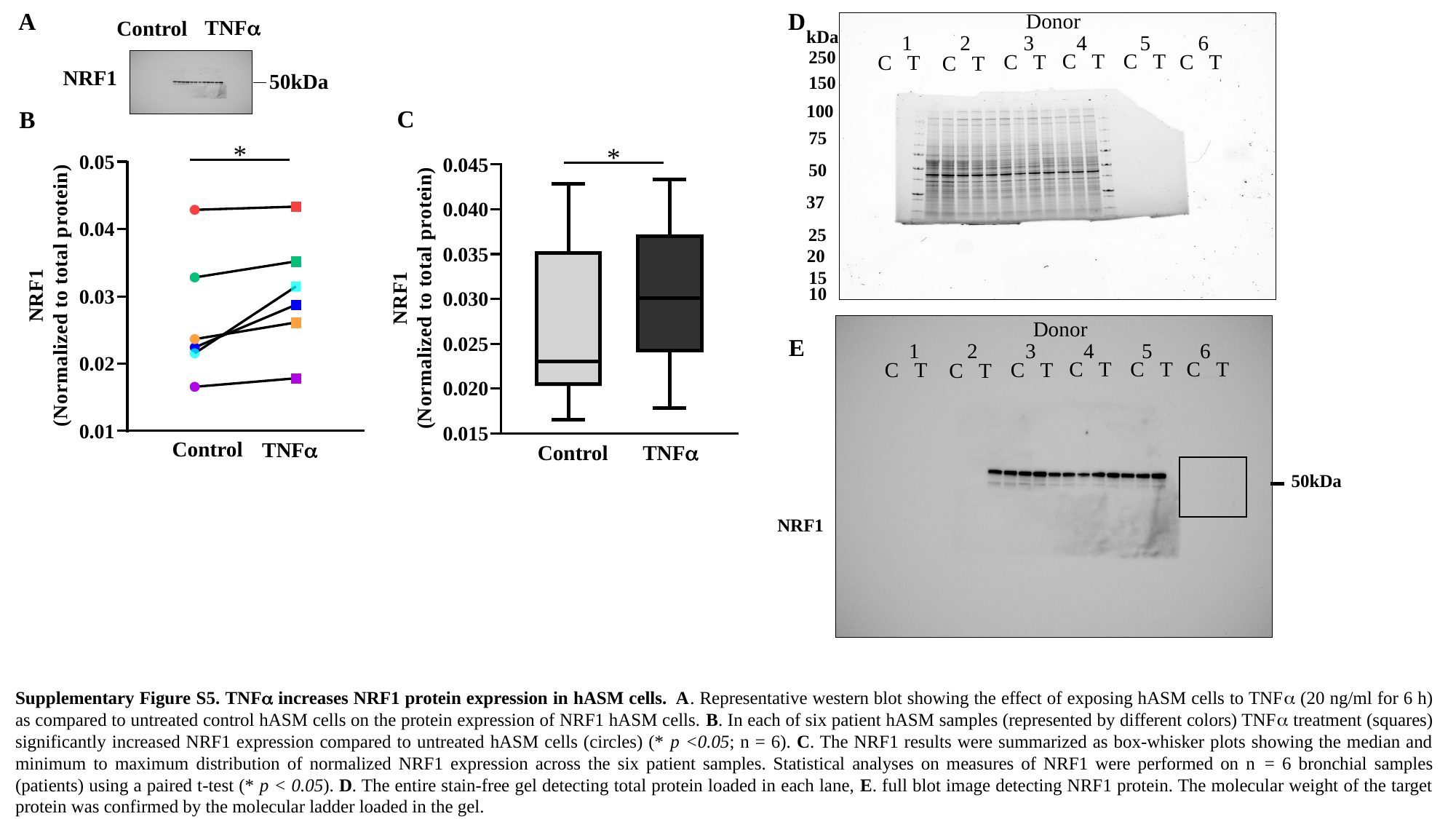

A
D
Donor
1 2 3 4 5 6
C T
C T
C T
C T
C T
C T
kDa
250
150
100
75
50
37
25
20
15
10
TNFa
Control
NRF1
50kDa
C
B
Control
TNFa
Control
TNFa
Donor
50kDa
NRF1
E
1 2 3 4 5 6
C T
C T
C T
C T
C T
C T
Supplementary Figure S5. TNFa increases NRF1 protein expression in hASM cells. A. Representative western blot showing the effect of exposing hASM cells to TNFa (20 ng/ml for 6 h) as compared to untreated control hASM cells on the protein expression of NRF1 hASM cells. B. In each of six patient hASM samples (represented by different colors) TNFa treatment (squares) significantly increased NRF1 expression compared to untreated hASM cells (circles) (* p <0.05; n = 6). C. The NRF1 results were summarized as box-whisker plots showing the median and minimum to maximum distribution of normalized NRF1 expression across the six patient samples. Statistical analyses on measures of NRF1 were performed on n = 6 bronchial samples (patients) using a paired t-test (* p < 0.05). D. The entire stain-free gel detecting total protein loaded in each lane, E. full blot image detecting NRF1 protein. The molecular weight of the target protein was confirmed by the molecular ladder loaded in the gel.
